# Characteristics of burn deaths from 2003 to 2009 in a burn center: A retrospective study

**DOI:** 10.4103/2321-3868.118933

**Published:** 2013-09-18

**Authors:** Jian Chen, Hong Yan, Gaoxing Luo, Qizhi Luo, Xiaolu Li, Jiaping Zhang, Zhiqiang Yuan, Daizhi Peng, Yizhi Peng, Jianian Hu, Jun Wu

**Affiliations:** Institute of Burn Research, Southwest Hospital, State Key Laboratory of Trauma, Burns and Combined Injury, The Third Military Medical University, Chongqing, China

**Keywords:** Burns, death, multiple organ dysfunction syndrome

## Abstract

Mortality remains one of the most important end-point quality control parameters to evaluate a burn care system. We retrospectively reviewed the characteristics and multiple organ dysfunction syndrome (MODS) patterns of burn deaths in our center from January 2003 to December 2009. The mortality rate during this time period was 2.3%. Fifty-six patients died, including 49 males and 7 females. The mean survival time was 28.45 ± 24.60 days. The burn percentage was (76.70 ± 26.86) % total burn surface area (TBSA), with (27.74 ± 24.95) % deep-partial thickness burns and (46.88 ± 33.84) % full-thickness burns. Inhalation injury was diagnosed in 36 (64.29%) patients. Patients who had undergone an operation, particularly in the first week post-burn, had a significantly longer survival time. An average of 5.50 ± 1.35 malfunctioning organs per patient and a mean sequential organ failure assessment (SOFA) score of 13.91 ± 3.65 were observed. The most frequently malfunctioning organs were involved in the respiratory, hematologic, circulatory, and central nervous systems. Most of the organ damage occurred during the first week post-burn, followed by 4 weeks later, with relatively less organ damage observed in the third week. Among patients with a TBSA over 50%, non-survivors had larger burn sizes (particularly larger full-thickness burns) and a higher incidence of inhalation injury compared with survivors; non-survivors were also more likely to have microorganism-positive blood and sputum cultures. In conclusion, burn deaths are related to a higher burn percentage, inhalation injury, MODS, and infection. Early operation may help improve survival duration.

## Introduction

In china, the incidence of burn injury remains high because of the large population and the deficiency in safety education. However, the nationwide statistical data on incidence and mortality are not officially available, and few countries have reported comparable data. Panjeshahin *et al.*, reported incidence rates ranging from 4.6 to 120 burns per 100,000 person-years and mortality rates of 0.23–4.6 burns per 100,000 person-years in different countries.[1]

**Table:** 

Access this article online	
**Quick Response Code:**	**Website:** www.burnstrauma.com
	**DOI:** 10.4103/2321-3868.118933

The mortality rate remains one of the most important end-point quality control parameters to evaluate a burn care system. During the past 50 years, the mortality rate of burn inpatients in our center decreased remarkably from 12.80% in 1958–1968 to 2.03% in 1986–2005.[2] This improvement could be attributed to the establishment of specialized burn care unit, therapeutic improvements in first aid pre-admission, prompt and adequate fluid resuscitation, infection control, early wound excision and closure, nutrition support, organ function protection, and the innovation of new antibiotics.[3]

Although the overall mortality rate remarkably decreased, great difficulties remain in the treatment of severe burn patients.[3] Therefore, a retrospective study was conducted to analyze the characteristics of burn deaths at our center and to attempt to identify the most influential factors that may help to improve burn care.

## Materials and methods

### Setting

The Institute of Burn Research of Southwest Hospital is one of the largest burn centers in China. It has 150 inpatient beds, and over 1000 patients have been admitted annually in recent years, including patients with burns, trauma, chronic wounds, and decubital ulcers, and patients requiring plastic surgery. Most patients reside in Chongqing (one of the biggest cities of Southwest China, with a population of 30 million) and some adjacent provinces such as Sichuan, Guizhou, and Yunnan.

We retrospectively reviewed the records of inpatients in our burn center from January 2003 to December 2009; 56 burn patients died during hospitalization, and the total number of burn inpatients was 2434. Therefore, the inpatient mortality rate during this time period was 2.30%. Patient demographics (age, sex, burn size, and depth of burn), the incidences of inhalation injury and sepsis, the sequential organ failure assessment (SOFA) score, and mortality were recorded.

The SOFA score was used to assess the severity of multiple organ dysfunction syndrome (MODS), which was defined as having a minimum of two organs involved with a total score greater than 4.[4]

### Burn management

Standard treatment protocols in the burn center of Southwest Hospital from 2003 to 2009 included fluid resuscitation with a balanced solution and plasma according to the Third Military Medical University (TMMU) formula.[5] Briefly, the TMMU formula indicates the following: For adult burn patients, the coefficient of fluid resuscitation was 1.5 ml (1 ml crystalloid solution + 0.5 ml colloid) per 1% total burn surface area (TBSA) per kg of body weight (BW) in the first 24 h and one-half of the actual infused volume of crystalloid solution and colloid in the second 24 h. In addition, 2l of water (using a 5% glucose solution) was added as a daily basic requirement. Partial-thickness burn wounds of less than 20% TBSA were covered with dressings; burns with greater areas of damage and full-thickness burns were initially treated with silver sulfadiazine. For full-thickness burns, primary excision and biological closure with autografts, allografts, or both were started on the third day post-burn.

Patients who were exposed to smoke or fire in a closed space or had a deep burn on the face or neck were suspected to have inhalation injury, which was confirmed in the presence of signs of hoarse voice, dyspnea, or carbon particles in sputum or by bronchoscopy. Incision of the trachea and intubation were carried out in the presence of airway obstruction.

Intravenous antibiotics were routinely prophylactically administered to burn patients with wounds of greater than 20% TBSA. Antibiotics were used therapeutically in the presence of clinically suspected or confirmed infection and adjusted according to the results of microorganism culture.

Nutritional requirements were calculated using a modified Curreri formula; enteral feeding and parenteral nutrition were provided based on the patient’s condition.

### Data analysis

Data regarding age, gender, burn size, survival time, inhalation injury, operation, etc. were collected and analyzed. The change in assessment criteria was evaluated using a *t*-test of independent samples to compare means between two continuous variables, and the Chi-square test was used to compare percentages. Analyses were performed in SPSS 10.0 software. *P*≤ 0.05 was considered significant.

## Results

### Demographics of the burn deaths from 2003 to 2009 in our center

**Figure 1:**
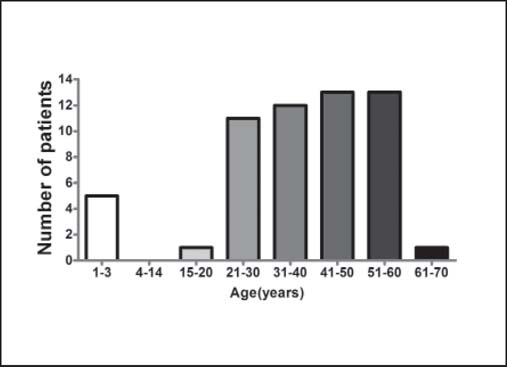
The age distribution of non-survivors in our center from 2003 to 2009.

**Figure 2:**
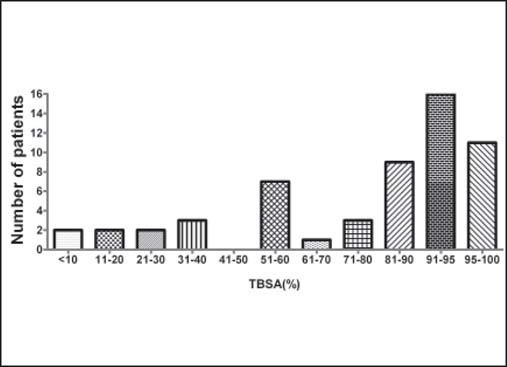
The distribution of TBSA for non-survivors in our center from 2003 to 2009.

A total of 56 patients, including 49 males and 7 females, died during the observational period. The mean age was 37.33 ± 15.64 years [Figure 1]. The causes of injury were as follows: 26 (46.4%) scalds, 10 (17.8%) flame burns, 9 (16.1%) thermal cement burns, 9 (16.1%) explosions, and 2 (3.6%) electrical burns. The mean survival time was 28.45 ± 24.60 days. Five of the 56 patients died on the day of injury, 2 of whom were children (a 1-year-old baby with a 10% TBSA burn on the head and a 3-year-old child with a 30% TBSA burn); the other 3 were adults with burn sizes of 96%, 99%, and 99% TBSA, respectively. The mean TBSA was (76.70 ± 26.86)%; including (27.74 ± 24.95)% deep-partial thickness burns and (46.88 ± 33.84)% full-thickness burns [Figure 2]. Inhalation injury was diagnosed in 36 (64.29%) patients.

### Survival time of non-survivors

**Figure 3:**
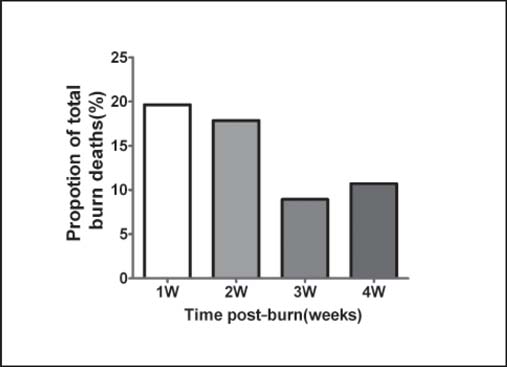
The distribution of burn deaths in the first 4 weeks post-burn in our center.

The mean survival time was 28.45 ± 24.60 days. The distribution is shown in Figure 3. During the first month post-burn, the number of burn deaths was highest in the first week, followed by the second week. Nearly 42.83% of burn deaths occurred 4 weeks post-burn. For patients with greater than 80% TBSA, a similar time distribution was observed. However, patients with greater than 90% TBSA had a significantly shorter survival time (26.55 ± 21.61 days) compared with patients with 80–89% TBSA (43.40 ± 32.59 days).

### The relationship between survival time and operation

In total, 41 patients were operated on at least once before death and 25 were operated on within the first week post-burn. Patients who underwent an operation, particularly in the first week post-burn, had a significantly longer survival time [Table 1].

**Table 1:** The relationship between survival time and operation

	Time of operation (days post-burn)	Survival time (days)	Patient number
Operated	1–49	37.11±24.97*	41
	1–7	34.28±22.86*	25
	8–14	21.33±10.91	6
Non-operated		18.67±15.64	15

*P < 0.05, vs. non-operated

### Organ dysfunction and burn death

**Figure 4:**
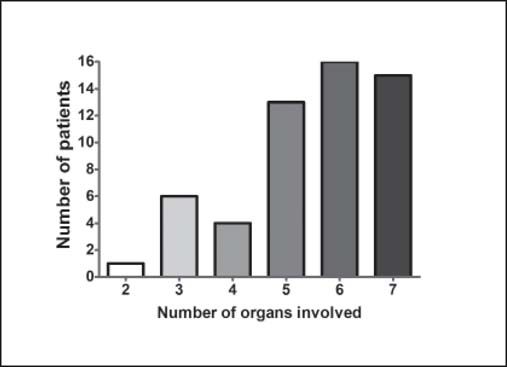
The number of dysfunctioning organs involved in burn deaths.

According to the SOFA score, all of the patients who died had a minimum of two organs involved, with a total score greater than 4. The number of organs involved in these patients is shown in Figure 4. We observed an average of 5.5 ± 1.35 organs involved per patient and a mean SOFA score of 13.91 ± 3.65.

The most frequently affected organs were involved in the respiratory, hematological, circulatory and central nervous systems (CNS) [Table 2]. Most organ damage occurred during the first week post-burn, followed by 4 weeks post-burn, and less organ damage was observed in the third week. the severity of organ damage was as follows: respiratory > hematology > circulation > CNS > renal > digestive system > liver. The order of onset of organ system damage was as follows: hematology > respiratory > renal > liver > digestive system > CNS > circulation [Table 3].

**Table 2:** Organ dysfunction by weeks post-burn

Systems and organs involved	Patient number (*N*=56)	Time post-burn (weeks)			
		<1	1–2	3–4	>4
Circulation	50 (89.29%)	20	9	5	16
Respiratory	53 (94.64%)	40	4	2	7
Central nervous system	49 (87.50%)	19	11	5	14
Hematology	49 (87.50%)	39	2	2	6
Liver	32 (57.14%)	22	0	5	5
Renal	37 (66.07%)	26	3	2	6
Digestive system	37 (66.07%)	14	12	2	9

**Table 3:** Time of onset and severity of organ dysfunction in the burn deaths

Organ involved	Time of onset (days post-burn)	SOFA score
Circulation	20.42 ± 21.45	2.82±0.56
Respiratory	9.00 ± 13.69	3.09±0.71
Central nervous system	17.94 ± 18.92	2.42±0.84
Hematology	8.84 ± 14.01	2.88±0.44
Liver	11.63 ± 15.90	1.69±0.54
Renal	11.08 ± 16.64	2.38±1.01
Digestive system	17.32 ± 17.51	1.89±0.97

### Demographics of patients with a burn size over 50% TBSA

From 2003 to 2009, the mortality of patients with a burn size ≤50% TBSA was only 0.39%, whereas it reached 32.19% among patients with a burn size over 50% TBSA. This suggests that a large burn size greatly impacts burn patient mortality. The demographic characteristics of surviving and non-surviving patients with over 50% TBSA in our center from 2003 to 2009 are compared in Table 4.

**Table 4:** Demographics of patients with a burn size over 50% TBSA from 2003 to 2009

	Non-survivors (*n*=47)	Survivors (*n*=99)
Male	41 (87.23%)	73 (73.74%)
Female	6 (12.77%)	26 (26.26%)
Age (years)	39.42±12.76	36.59±17.78
Scald	21 (44.68%)	20 (20.20%)
Flame burn	8 (17.02%)	58 (58.59%)
Thermal cement burn	9 (19.15%)	4 (4.04%)
Explode	8 (17.02%)	15 (15.15%)
Electrical burns	1 (2.13%)	2 (2.02%)
Mean total burn surface area	86.34 ± 14.80*	71.03 ± 13.61
Mean area of deep-partial thickness burn	31.11 ± 25.66	36.08 ± 20.54
Mean area of full-thickness burn	52.70 ± 32.49*	28.36 ± 24.47
Inhalation injury	33 (70.21%)*	52 (52.53%)

*P < 0.05 non-survivors vs. survivors

Non-survivors had a larger burn size, particularly larger areas of full-thickness burns. The incidence of inhalation injury was much higher in non-survivors than in survivors.

### Infection in burn patients with over 50% TBSA

For patients with a burn size over 50% TBSA, non-survivors had higher incidences of microorganism-positive blood and sputum cultures. However, wound cultures did not show significant differences [Table 5].

**Table 5:** Culture positivity of samples from burn patients with over 50% TBSA

Burn size	Non-survivors	Survivors
Samples	(*n*=47)	(*n*=99)
Blood	20 (42.55%)*	8 (8.08%)
Wound	33 (70.21%)	64 (64.65%)
Sputum	29 (61.70%)*	46 (46.46%)

*P < 0.05 vs. survivors

## Discussion

The mortality rate remains one of the most important end-point quality control parameters to evaluate a burn care system. the reported mortality rate of inpatients varies among countries.[6] In western countries, the overall mortality rate is 5.00–6.00%,[7,8] whereas in our center, the reported mortality rate was 2.03%. Xiao and Cai’s study was conducted on 5321 burn patients hospitalized in a burn center in Jinzhou, China, from 1980 to 1998, and their overall mortality rate was only 0.86%. The author indicated that the high survival rate may have been related primarily to the low percentages of older patients and patients with severe burns.[9] A retrospective study including 1974 burn patients admitted to Jishuitan Hospital in Beijing was conducted from 2000 to 2008, and the data show a mortality rate of 2.8%, which is similar to our observation.[10] Another retrospective study by Li, one of the founders of the burn care system in China, reported patient data from 1958 (the start of the modern burn care system in China) to 1998 in burn centers in military hospitals in China. In his report, the mortality rates were 4.93% (48,978 patients at 16 centers) from 1958 to 1979, 5.21% (64,320 patients at 29 centers) from 1980 to 1992, and 3.81% (48,085 patients at 28 centers) from 1993 to 1998; our data show further improvement.[3]

The mean survival time was 28.45 ± 24.60 days, and nearly half of the patients survived the first 4 weeks post-burn. Pruitt reported a survival time of 17 days in 1970–1971 and 24 days in 1986–1987.[11] Krishnan *et al.*, reported a length of stay in cases of burn deaths of 26.4 ± 45.0 days, with an average TBSA of 43.7 ± 26.8% and an average age of 52.9 ± 19.4 years.[12] Our data show further improvement in burn care.

MODS remains the leading cause of death among severe burn patients.[13–15] Cumming *et al.*, reported that with >20% TBSA, the rate of MODS reached 63.4% and that 28.0% of these cases presented severe MODS.[16] in our study, non-survivors had at least two or more dysfunctioning organs, with 5.50 ± 1.35 affected organs per patient and an average SOFA score of 13.91 ± 3.65.

Among these compromised organs, the respiratory system was most frequently involved, and it was the earliest affected and typically the most severely damaged. This is in line with the results of several other studies.[17,18] In our study, 64.29% of patients had confirmed inhalation injury and 55.45% had pulmonary infections; however, 94.64% of patients presented respiratory dysfunction, which suggests that other factors in addition to inhalation injury and infection are involved. Steinvall *et al.*, reported that Acute Respiratory Distress Syndrome (ARDS) induced by burn-induced inflammation and not secondary to sepsis could also contribute to respiratory dysfunction.[19]

Our data indicate that the hematologic system (using platelet counts as an index) was also affected. There was a high incidence of hematologic dysfunction, with an early onset post-burn, and it demonstrated the most severe changes. Even so, the relationship between blood platelet (PLT) counts and mortality was not clear, as few patients died of hemorrhage as a result of low PLT levels. Choctaw and Michas first reported decreases in the platelet counts of 13 patients with severe burns after the initial period of burn shock and that it took 7–12 days for their platelet levels to return to within the normal range.[20] Takashima attempted to divide the fluctuation of PLT counts into three stages: the early decrease in PLTs due to consumption, the synthesis of new PLTs by the bone marrow, and the fluctuating phase of PLT maturation. The platelet behavior during this fluctuating phase reflected a consumptive coagulopathy driven by intermittent endotoxemia or other physiological disturbances.[21] Guo *et al.*, found that an initial slump of PLTs, particularly a percentage decline of the platelet count of 65% or greater during the first 3 days, provided prognostic significance for 30-day mortality in severely burned patients.[22] In our practice, the extent and duration of the PLT decrease always appeared to be associated with the infection status and severity of the burn. Further observations and discussion of this association are required.

Organ dysfunction occurred at any time following the burn injury, but the incidences in the first and fourth weeks were much higher, which is consistent with the survival distribution of the non-survivors [Figure 3]. However, this is different from the reports of other authors, who have noted MODS occurrence primarily during the second week post-burn.[23] However, the larger burn sizes observed in our cases may account for this difference. For this group of patients, the current model of early fluid resuscitation might not meet the real needs of the internal organs[24] and may lead to early organ damage and death.

Many scholars agree that improvements in the prevention and control of burn infection may decrease the morbidity and mortality of MODS. For severe burn patients, the long-standing wounds and frequent invasive procedures, such as arterial or venous catheterization, tracheotomy, Continuous Renal Replacement Therapy (CRRT), and artificial ventilation, lead to a high risk of infection.[25–27] Our data demonstrated that non-survivors more often had positive blood cultures and pulmonary infection. The high risk and incidence of infection were the most important factors related to the occurrence of MODS. Sharma *et al.*, reported that in 65% of fatal burn cases, septicemia was the cause of death in their autopsy study[28] Moreover, another report concluded that 75% of all deaths following burns are related to infection.[29]

The mortality rate increased dramatically when the TBSA was larger than 50%. However, even in this group of patients, they still had the chance to survive. Sepsis (diagnosed by a positive blood culture) and MODS were the most common causes of death. Indeed, it is somewhat frustrating that the predominant bacteria in burn wards are becoming more and more intractable. When infection cannot easily be treated due to a shortage of effective antibacterial drugs, it is more important to eliminate infection sources.[30–34] Our data indicate that patients who received surgery had a longer survival time than those who did not. Furthermore, operations performed during the first 7 days post-burn significantly lengthened survival time. Therefore, the early and adequate removal of burned tissue and effective coverage appear to be fundamental in burn management.

Early excision with grafting is an important step in ensuring survival.[35] However, it is challenging to use the limited amount of donor-site skin to cover large wounds. In China, cultured epidermal autografts and Integra™ are still not available. Microskin grafting has become one of the most important methods used for the treatment of extensive burns.[36–38] It may partially solve the problem associated with large wounds and limited donor sites, but is still far from ideal. The time of allograft rejection or detachment is not fully predictable and varies and may be affected by the donor’s age, the preservation method, time,[39] wound infection, and other factors that need to be further studied.

Treatment of severe burn patients is not only a great challenge to burn care systems but also brings heavy social and economic burdens to the families of burn victims and society. In recent years, our unit has been treating nearly 1500 inpatients each year and we have been always confronted with problems such as medical staffing shortages and the financial constraints of the patients. When we rethink the contradictions between the limited medical services and the large population of burn patients we encounter, it appears to be time for us to face the question of whether we should re-allocate our medical services and pay more attention to patients with more hope and a better predicted outcome. However, before any prudent decisions are made, more investigations and studies are required to clarify the factors that affect outcome.

## Conclusion

Burn deaths are related to the percentage of TBSA, inhalation injury, MODS, and infection. Early operation may help improve survival time.

## References

[1] Panjeshahin MR, Lari AR, Talei AR, Shamsnia J, Alaghehbandan R (2001). Epidemiology and mortality of burns in the South West of Iran. Burns.

[2] Luo G, Peng Y, Yuan Z, Liu Y, Cheng W, Huang Y (2010). Inhalation injury in southwest China — The evolution of care. Burns.

[3] Li A, Yang ZC, Li GR (1999). The analysis of 48085 burn patients. Med J Chin Peoples Liberation Army.

[4] Marshall JC, Cook DJ, Christou NV, Bernard GR, Sprung CL, Sibbald WJ (1995). Multiple organ dysfunction score: A reliable descriptor of a complex clinical outcome. Crit Care Med.

[5] Luo G, Peng Y, Yuan Z, Cheng W, Wu J, Tang J (2009). Fluid resuscitation for major burn patients with the TMMU protocol. Burns.

[6] Bloemsma GC, Dokter J, Boxma H, Oen IM (2008). Mortality and causes of death in a burn centre. Burns.

[7] Saffle JR, Davis B, Williams P (1995). Recent outcomes in the treatment of burn injury in the United States: A report from the American Burn Association Patient Registry. J Burn Care Rehabil.

[8] Miller SF, Bessey PQ, Schurr MJ, Browning SM, Jeng JC, Caruso DM (2006). National Burn Repository 2005: A ten-year review. J Burn Care Res.

[9] Xiao J, Cai B (2003). Mortality rates among 5321 patients with burns admitted to a burn unit in China: 1980–1998. Burns.

[10] Cheng W, Yan-hua R, Fang-gang N, Wei-li D, Guo-an Z (2012). Epidemiology of 1974 burn patients at a major burn center in Beijing: A 9-Year Study. J Burn Care Res.

[11] Pruitt BA (1990). Infection and the burn patient. Br J Surg.

[12] Krishnan P, Frew Q, Green A, Martin R, Dziewulski P (2013). Cause of death and correlation with autopsy findings in burns patients. Burns.

[13] Guo F, Chen X, Wang YJ, Wang F, Chen XY, Sun YX (2009). Management of burns of over 80% of total body surface area: A comparative study. Burns.

[14] Wolf SE, Prough DS, Herndon DN, Herndon DN (2002). Aetiology and prevention of multisystem organ failure. Total burn care.

[15] Sheridan RL, Ryan CM, Yin LM, Hurley J (1998). Tompkins RG Death in the burn unit: Sterile multiple organ failure. Burns.

[16] Cumming J, Purdue GF, Hunt JL, O’Keefe GE (2001). Objective estimates of the incidence and consequences of multiple organ dysfunction and sepsis after burn trauma. J Trauma.

[17] Sheridan RL, Ryan CM, Yinb IL, Hurley J, Tompkins RG (1998). Death in the burn unit: Sterile multiple organ failure. Burns.

[18] Taran A, Baciu N, Rafulea V, German A (2005). Clinical and autopsy diagnoses of visceral affections of patiens who died because of complicated burns with multiple organ failure. Ann Burns Fire Disasters.

[19] Steinvall I, Bak Z, Sjoberg F (2008). Acute respiratory distress syndrome is as important as inhalation injury for the development of respiratory dysfunction in major burns. Burns.

[20] Choctaw WT, Michas C (1981). Comparative coagulation abnormality patters in moderately and severely burned patients. Burns.

[21] Takashima Y (1997). Blood platelets in severely injured burned patients. Burns.

[22] Guo F, Wang X, Huan J, Liang X, Chen B, Tang J (2012). Association of platelet counts decline and mortality in severely burnt patients. J Crit Care.

[23] Nguyen LN, Nguyen TG (2009). Characteristics and outcomes of multiple organ dysfunctiion syndrome among severe-burn patients. Burns.

[24] Dulhunty JM, Boots RJ, Rudd MJ, Muller MJ, Lipman J (2008). Increased fluid resuscitation can lead to adverse outcomes in major-burn injured patients, but low mortality is achievable. Burns.

[25] Fitzwater J, Purdue G F, Hunt JL, O’Keefe GE (2003). The risk factors and time course of sepsis and organ dysfunction after burn trauma. J Trauma.

[26] Soares dE Macedo JL, Santos JB (2006). Nosocomial infections in a Brazilian Burn Unit. Burns.

[27] Santucci SG, Gobara S, Santos CR, Fontana C, Levin AS (2003). Infections in a burn intensive care unit: Experience of seven years. J Hosp Infect.

[28] Sharma BR, Harish D, Singh V P, Bangar S (2006). Septicemia as a cause of death in burns: An autopsy study. Burns.

[29] Vindenes H, Bjerknes R (1995). Microbial colonization of large wounds. Burns.

[30] Rosenthal SR (1982). Burn toxin and its competitin. Burns Incl Therm Inj.

[31] Allgower M, Schoenenberger GA, Sparkes BG (1995). Burning the largest immune organ. Burns.

[32] Mosier MJ, Gibran NS (2009). Surgical Excision of the Burn Wound. Clin Plast Surg.

[33] Bone RC (1992). Toward an epidemiology and natural history of SIRS (systemic inflammatory response syndrome). JAMA.

[34] Bone RC, Sprung CL, Sibbald WJ (1992). Definitions for sepsis and organ failure. Crit Care Med.

[35] Herndon DN (2002). Total burn care.

[36] Zhang M, Zhou G, Zhang P (2001). Microskin grafting in recent 15 years. Zhonghua Wai Ke Za Zhi.

[37] Zhang ML, Chang ZD, Han X, Zhu M (1986). Microskin grafting. I. Animal experiments. Burns Incl Therm Inj.

[38] Zhang ML, Wang CY, Chang ZD, Cao DX, Han X (1986). Microskin grafting. II. Clinical report. Burns Incl Therm Inj.

[39] Franchini M, Zanini D, Bosinelli A, Fiorini S, Rizzi S, D’Aloja C (2009). Evaluation of cryopreserved donor skin viability: The experience of the regional tissue bank of Verona. Blood Transfus.

